# Biomechanical Loading Evaluation of Unsintered Hydroxyapatite/poly-l-lactide Plate System in Bilateral Sagittal Split Ramus Osteotomy

**DOI:** 10.3390/ma10070764

**Published:** 2017-07-07

**Authors:** Shintaro Sukegawa, Takahiro Kanno, Yoshiki Manabe, Kenichi Matsumoto, Yuka Sukegawa-Takahashi, Masanori Masui, Yoshihiko Furuki

**Affiliations:** 1 Division of Oral and Maxillofacial Surgery, Kagawa Prefectural Central Hospital, 1-2-1, Asahi-machi, Takamatsu, Kagawa 760-8557, Japan; tkanno@med.shimane-u.ac.jp (T.K.); kenichi.matsumoto.2.4@gmail.com (K.M.); yuka611225@gmail.com (Y.S.-T.); de18048@s.okayama-u.ac.jp (M.M.); furukiy@ma.pikara.ne.jp (Y.F.); 2 Department of Oral and Maxillofacial Surgery, Shimane University Faculty of Medicine, Shimane 693-8501, Japan; 3 Admission Center, Kagawa University, Takamatsu, Kagawa 760-0016, Japan; manabe@kms.ac.jp

**Keywords:** sagittal split ramus osteotomy, unsintered hydroxyapatite/poly-l-lactide composite plate, bioactive resorbable plate, biomechanical loading evaluation, tensile and shear strength evaluation

## Abstract

OSTEOTRANS MX^®^ (Takiron Co., Ltd., Osaka, Japan) is a bioactive resorbable maxillofacial osteosynthetic material composed of an unsintered hydroxyapatite/poly-l-lactide composite, and its effective osteoconductive capacity has been previously documented. However, the mechanical strength of this plate system is unclear. Thus, the aim of this in vitro study was to assess its tensile and shear strength and evaluate the biomechanical intensity of different osteosynthesis plate designs after sagittal split ramus osteotomy by simulating masticatory forces in a clinical setting. For tensile and shear strength analyses, three mechanical strength measurement samples were prepared by fixing unsintered hydroxyapatite/poly-l-lactide composed plates to polycarbonate skeletal models. Regarding biomechanical loading evaluation, 12 mandibular replicas were used and divided into four groups for sagittal split ramus osteotomy fixation. Each sample was secured in a jig and subjected to vertical load on the first molar teeth. Regarding shear strength, the novel-shaped unsintered hydroxyapatite/poly-l-lactide plate had significantly high intensity. Upon biomechanical loading evaluation, this plate system also displayed significantly high stability in addition to bioactivity, with no observed plate fracture. Thus, we have clearly demonstrated the efficacy of this plate system using an in vitro model of bilateral sagittal split ramus osteotomy of the mandible.

## 1. Introduction

The standard osteofixation in orthognathic surgery has been titanium osteosynthesis for many years [1,2]. Internal fixation devices have the benefits of securing the osteotomy segments, preventing displacement from muscular pull, shortening the healing period, obviating the need for maxillomandibular fixation, and preventing relapse [3]. Recently, a biologically inert resorbable plate system has been introduced to eliminate the need for a second operation for removing the fixation material. Although many previous reports have shown resorbable osteosynthesis to yield good clinical results, these platforms are not widely used due to their handling properties, intensity, and insecurity concerning their ability to maintain segments in the proper position [4]. 

Meanwhile, resorbable osteosynthesis technology is constantly evolving in its capacity to enhance bioresorbability and marked bioactive osteoconductivity, with new material compositions conferring different and improved in-situ behaviors. OSTEOTRANS MX^®^ (Takiron Co., Ltd., Osaka, Japan), also called Super FIXSORB MX^®^ in Japan, is a bioactive and totally resorbable maxillofacial osteosynthetic bone fixation material, which has been reported to exhibit clinical efficacy with relatively long-term results [5,6]. However, both the strength and stability of this plate system are still unclear. Thus, the aims of this in vitro study were to assess the physical strength of this bioactive resorbable plate system, as well as the biomechanical intensity of different osteosynthesis plate designs after sagittal split ramus osteotomy (SSRO) by simulating masticatory forces in a clinical setting. 

## 2. Materials and Methods

### 2.1. Materials

Forged composites of unsintered hydroxyapatite/poly-l-lactide (u-HA/PLLA), prepared via the same means as OSTEOTRANS MX^®^, were processed by machining or milling treatments into various miniscrews and miniplates, which, respectively, contained 30 and 40 weight fractions of u-HA (raw hydroxyapatite, neither calcined nor sintered material) particles in composites (hereinafter referred to as u-HA 30 miniscrew and u-HA 40 miniplate).

### 2.2. Tensile and Shear Strength Evaluation

#### 2.2.1. Sample Preparation

Mechanical strength measurement samples were prepared by fixing the plate with screws to the polycarbonate plate. The polycarbonate plates were fixed by different osteosynthesis methods using resorbable plate and screws to form the following groups:

- a single u-HA/PLLA straight plate, fixing the plate on each side with two screws (total four screws)
- double u-HA/PLLA straight plate, fixing the plate on each side with two screws (total eight screws)
- one u-HA/PLLA ladder plate, fixing the plate on each side with two screws (total eight screws)

We used all plates (thickness: 1.4 mm) and screws (diameter: 2 mm; length: 8 mm). The fixed models were mounted on autographs across the chuck. This test was measured on the maximum stress and the stress at the time of 1-mm movement until the plate or screw was destroyed, and the load was applied at a test speed of 10 mm/min. Two types of strength tests (the tensile and shear strength test) were performed, each in triplicate.

#### 2.2.2. Strength Tests

**Figure 1:**
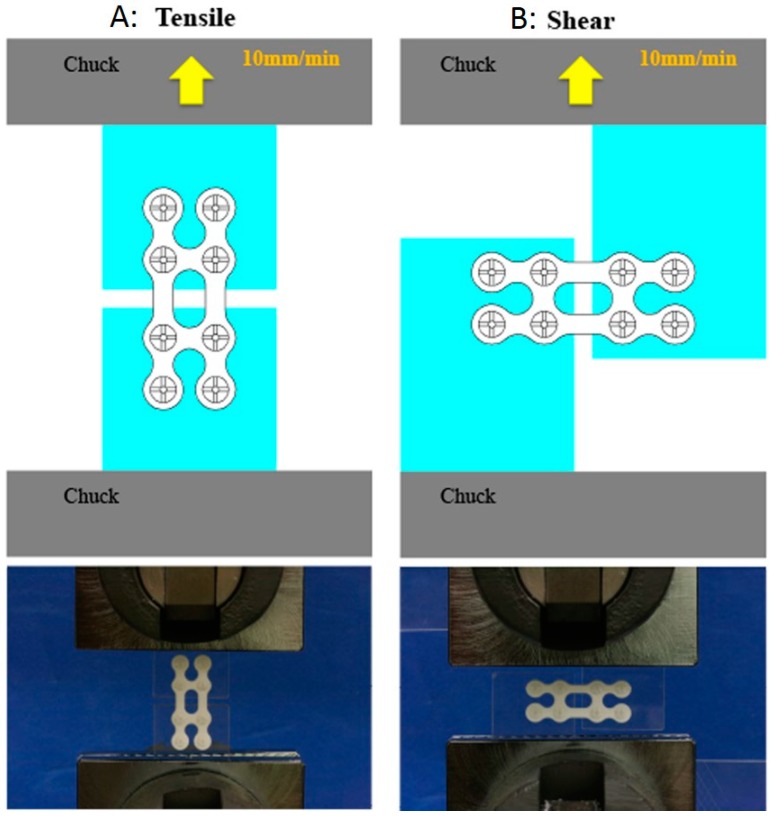
Mechanical strength models were prepared by fixing the plate with screws to the polycarbonate plate. (**A**). Tensile strength; (**B**). Shear strength.

Tensile strength (St) was measured by the method illustrated in Figure 1A according to the Japanese Industrial Standard (JIS) K7113 [7]. The peak value of the profile attained by an Autograph AGS 2000 D (Shimadzu Co., Kyoto, Japan) was considered the St. The temperature and relative humidity were 23 °C and 50%, respectively.

Shear strength (Ssp) was measured by the method illustrated in Figure 1B according to JIS K7113. The peak value of the profile attained by the Autograph AGS 2000 D was considered the Ssp.

### 2.3. Biomechanical Loading Evaluation

#### 2.3.1. Sample Preparation

**Figure 2:**
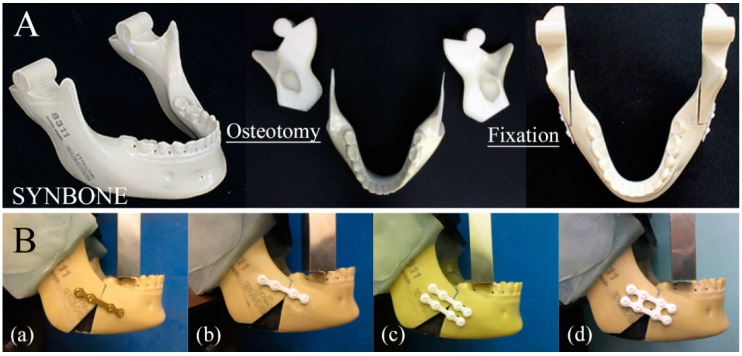
(**A**) Sagittal split ramus osteotomy mimicking the Dal Pont modification, guided by a computer-controlled program, was performed in the mandible. The buccal cortex osteotomy of the mandible model was carried out towards the angle of the mandible from the second molars. An additional bone model was prepared from bone defects so as not to be affected by bone interference; (**B**) (a) Single conventional titanium plate; (b) Single u-HA/PLLA straight plate; (c) Double u-HA/PLLA straight plates; (d) u-HA/PLLA ladder plate.

This study involved 12 polyurethane replicas of human mandibles with bonelike consistency, with a medullar and a cortical portion (Code #8311, SYNBONE AG, Laudquart, Switzerland). SSRO mimicking the Dal Pont modification, as guided by a computer-controlled program, was performed in the mandible. The buccal cortex osteotomy of the mandible model was carried out towards the angle of the mandible from the second molars. Further bone models were prepared from bone defects so as to not be affected by bone interference [8] (Figure 2A). The bone segments were fixed by different commercially available osteosynthesis methods using a titanium miniplate/resorbable plate and monocortical screws. In all groups, bone fixation could be performed without plate bending; thus, the following groups were formed (Figure 2B):

(a) a single conventional titanium straight plate (Synthes (Oberdorf, Switzerland) Compact Lock 2.0: 1.5 mm) with four screws (2.0 mm diameter × 6 mm long monocortical screws) was installed in each bone segment
(b) a single u-HA/PLLA straight plate (OSTEOTRANS MX^®^; thickness: 1.4 mm) with four screws (2.0 mm diameter × 6 mm long monocortical screws) was installed in each bone segment
(c) double u-HA/PLLA straight plates (OSTEOTRANS MX^®^; thickness: 1.4 mm), each with four screws (2.0 mm diameter × 6 mm long monocortical screws), were installed in each bone segment
(d) one u-HA/PLLA ladder plate (OSTEOTRANS MX^®^; thickness: 1.4 mm) with eight screws (2.0 mm diameter × 6 mm long monocortical screws) was installed in each bone segment.

#### 2.3.2. Loading Test

**Figure 3:**
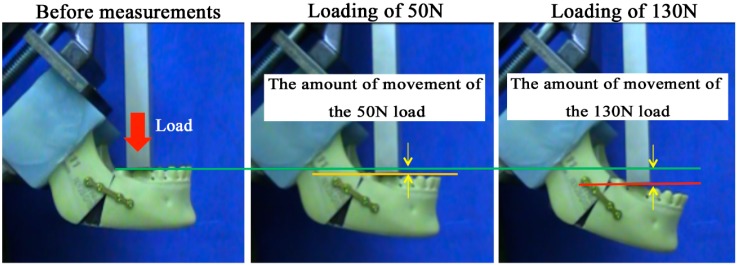
A linear load in the mandibular first molar region was applied at a displacement speed of 10 mm/min. We compared the amount of movement at the time of the load in the postoperative average occlusal force (postoperative 1 week, about 50 N, and postoperative 1 month, about 130 N) to the reference.

After fixation, the specimens were mounted on a testing machine (AG-2kNXD, Shimazu, Japan), which was based on a biomechanical cantilever-bending model that simulates masticatory forces, and stabilized in the condylar and coronoid areas. An initial load was applied to standardize the test requirements, and the machine was then reset. A linear load in the mandibular first molar region was applied to the mandibles at a displacement speed of 10 mm/min. The resistance forces needed to displace the distal segment were transmitted from the load cell to a computer. During the maximum stress, it was difficult to compare the fixing condition for receiving the influence of such elongation of the plate and not only breakage of the plate. Therefore, we compared the amount of movement at the time of the load in the postoperative average occlusal force in reference to [9] (postoperative 1 week: 50 N; postoperative 1 month: 130 N) (Figure 3).

### 2.4. Statistical Analysis

To compare the amount of movement based on the fixed material under certain conditions, we performed a Tukey–Kramer test. JMP 11.0 for Mac computers was the statistical software package used (SAS Institute Inc., Cary, NC, USA). *p* < 0.05 was considered statistically significant.

## 3. Results

### 3.1. Tensile and Shear Strength Evaluation

The results shown in Table 1 and Table 2 indicate that the u-HA/PLLA ladder plate group was significantly superior to other plate systems with regards to St and Ssp. In this study, all breakages were plates; there was no screw breakage.

#### 3.1.1. Tensile Strength 

**Figure 4:**
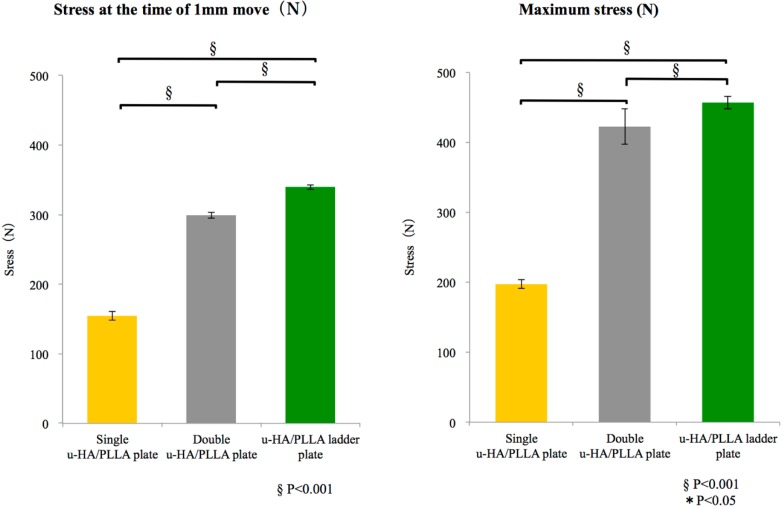
Mean values and respective standard deviations for the tensile strength test for each group. Left graph shows the maximum stress, and the right graph shows the stress during 1-mm movement in tensile strength.

There was a significant difference among groups, as well as between the single u-HA/PLLA plate and double u-HA/PLLA plate groups and between the double u-HA/PLLA plate and u-HA/PLLA ladder plate groups. In particular, the u-HA/PLLA ladder plate group was significant regarding stress at the time of 1-mm movement and the maximum stress (Figure 4).

#### 3.1.2. Shear Strength

**Figure 5:**
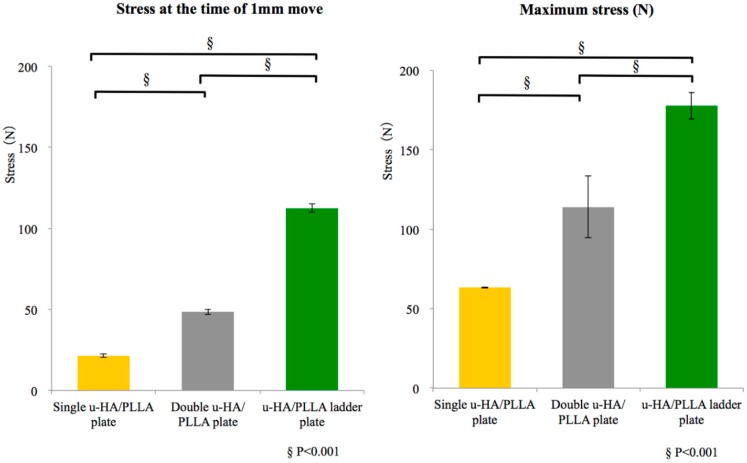
Mean values and respective standard deviations for the shear strength test for each group. Left graph shows the maximum stress, and the right graph shows the stress during 1-mm movement in shear strength.

The comparison among groups showed a significant difference between the u-HA/PLLA ladder plate and single and double u-HA/PLLA plate groups regarding stress at the time of 1-mm movement and the maximum stress. The u-HA/PLLA ladder plate group had a significantly higher mechanical strength than any other group (Figure 5).

### 3.2. Biomechanical Loading Evaluation

**Figure 6:**
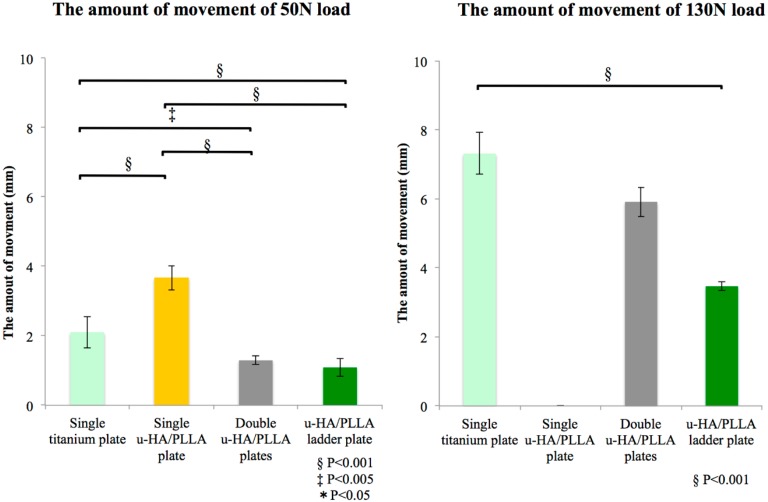
Mean values and respective standard deviations for the amount of movement by adding load for each group. Left graph shows the amount of movement with 50 N load, and the right graph shows the amount of movement with 130 N load.

The titanium plate and single u-HA/PLLA straight plate groups deformed, and none of the plates were broken or fractured with a 50 N load. With a 130 N load, all plates in the single u-HA/PLLA group were broken. However, only one sample in the double u-HA/PLLA group fractured upon the 130 N load. The titanium plate group was accompanied by movement of large bone fragments due to the deformation of plates at 130 N load. Although the u-HA/PLLA ladder plate group moved bone fragments a little, none of the plates were broken. The single u-HA/PLLA plate group significantly moved more, compared to all the other osteosynthesis materials. This group could not be compared to the broken plates in the single and double u-HA/PLLA groups. On the other hand, the titanium plate group exhibited significantly greater movement than the u-HA/PLLA ladder plate group (Table 3, Figure 6).

## 4. Discussion

An overwhelming majority of orthognathic surgery patients ask for a resorbable osteosynthesis system [10,11,12]. However, a resorbable fixation plate is, on the whole, weaker than titanium and produces adverse effects in vitro [13]. Although multiple resorbable plates and screws are put in place to enhance strength, the resorbable osteosynthesis system might fracture as a result of the excessive occlusal force imposed by physical properties of the material. The rigidity and stability between fractured bone segments promoted by an osteosynthesis method are the primary factors in patient recovery, because if bone healing is not efficient, resorbable osteosynthesis methods may cause serious impairments to treatment [14]. Therefore, there is an increased interest in developing a more adequate osteosynthesis method that is bioactive with minimal morbidity.

OSTEOTRANS MX^®^, the bioactive and resorbable osteoconductive plate system used in this study, is made from a composite of uncalcined u-HA/PLLA. Use of this resorbable plate system obviates the need for a second surgery to remove the plate. Moreover, it has been clinically applied in various maxillofacial surgeries, such as those for trauma, fractures [15,16], and reconstruction [17]. Resorbable plates have been previously made from PLLA alone. However, PLLA osteosynthetic devices have several disadvantages, including lower dynamic strength, an inability to fuse with bone, and long resorption and replacement times [18,19,20,21]. OSTEOTRANS MX^®^ was designed to overcome these problems, and the u-HA/PLLA composite material was developed by adding particulate resorbable uncalcined and unsintered HA to PLLA as aforementioned. Shikinami et al. [22] reported that HA crystals can bind directly to bone in vivo. In addition, it has been reported that the plates directly bonded to bone clearly displayed the effective osteoconductivity of the u-HA/PLLA plate system in maxillofacial regions [15,16]. Its early osteoconductive bioactivity can be advantageous for early functional improvement after orthognathic surgery. Because of its bioactive, osteoconductive, as well as bioresorbable properties, the u-HA/PLLA composite fixation system has immense potential and clinically advantageous and may broaden its applicability in various aspects of orthognathic surgery as a feasible next generation material.

Regarding tensile stress, both the maximum stress and the stress at the time of 1-mm movement of the double u-HA/PLLA and u-HA/PLLA ladder plate groups were significantly higher than the single u-HA/PLLA plate group. Although the number of screws across the osteotomy line was the same for the two groups, there were only two screws along the tensile direction for the single u-HA/PLLA plate group. On the other hand, because four screws fixed the double u-HA/PLLA and u-HA/PLLA ladder plate groups, these results suggested high tensile strength without stress concentration to the screw hole periphery. For shear stress, both the maximum stress and the stress at the time of 1-mm movement in the u-HA/PLLA ladder plate group was significantly higher than in the single and double u-HA/PLLA plate groups. In the shear test, the plate tends to rotate about the screw hole. Since the u-HA/PLLA ladder plate was connected at a right angle, this resulted in interference with respect to rotational movement. In addition to the vertical connection in the u-HA/PLLA ladder plate, this plate system demonstrated higher stress possibly due to 3-dimentional stress distribution functional, together with preventing rotational movement via the distal screw fixation. In this study, we further confirmed the stability of the 3-dimentional plating system was sufficient in the u-HA/PLLA ladder plate group, consistent with that in similar experimental studies on biomechanics of titanium plate systems [23,24,25]. Furthermore, with regard to operability, because the distal screw is not vertically fixed, the consistency of the plate itself can be utilized. Therefore, it is unnecessary to bend plates, which could be fixed in close contact with bone fragments. This is an excellent system that combines strength and practicality.

Many biomechanical tests have been performed to evaluate the various fixation methods used for SSRO. To simulate clinical conditions, fresh sheep mandibles [26,27], synthetic polyurethane jaw models [23], and finite element models [24] have been utilized to determine the best human mandible fixation technique. However, according to past literature, the ideal material for biomechanical loading tests is human mandibular bone [25]. Since it is difficult to obtain this material due to legal and ethical reasons, such in vitro alternative models are controversial, as they cannot exactly reproduce the function of the human bone [28]. In our study, synthetic jaw models were used because they are easy to obtain, inexpensive, allow for standardization [28,29], and are amenable in evaluating the mechanical characteristics of fixation materials prior to their application in humans [27]. 

In this study, the biomechanical test was performed using a two-point model, which has been utilized in numerous studies for comparing the different osteosynthesis systems [23,25,27]. Since the weakness of this model may be that it does not accurately simulate the masticatory muscles, it has been previously suggested to use a three-point mechanical test [26]. However, Ribeiro-Junior et al. [30] believed that both two- and three-point models were poor substitutes for evaluating the fixation systems used in mandibular sagittal osteotomies due to their inability to truly reflect human mandibular function. Nevertheless, our experiments were performed using a two-point model, and we used the bilateral mandibular model to approximate the human condition without a hemimandibular model.

Currently, the trend for fixation after an SSRO is to use titanium miniplates with monocortical screws [31,32]. During the fracture healing period, premature failure of the plates must be prevented. The loads transmitted through the plates should not exceed the limit of strength of the material [33]. Bending of the bone plate for SSRO surgery, particularly in a resorbable plate system, has not been widely demonstrated. In our study, one of the experimental resorbable plates did not withstand a 50 N load. On the other hand, fixation of two plates had higher strength, compared to a one plate fixation. In previous biomechanical investigations, the parallel position of the two plates confers enhanced strength for SSRO fixation [34]. The double miniplates as a scheme of a two-point fixation largely enhance stability and decrease latent failure since stress is distributed over the two plates [31,32]. However, in the double resorbable plate fixation, large bone fragmental movement caused plate fractures in one sample without sufficient strength for a 130 N load, although the other two samples bore the movement. In addition, the u-HA/PLLA ladder plate showed a much higher strength than the titanium plate and double resorbable plate fixation. For the u-HA/PLLA ladder plate, its two arms hold the two fixation sites via a double step, similar to that in the 3-dimensional plating system. This allows a double miniplate-like action through a single construct with stress distributed over two fixation sites. In addition to the vertical connection in the u-HA/PLLA ladder plate, as shown by our shear test results, this plate system had higher stress due to preventing rotational movement via distal screw fixation. We have shown that such a plate system is a biomechanically effective plate structure for SSRO surgery.

This study will significantly contribute to the understanding of the biomechanical actions involved in chewing, loading, and movement by simulating masticatory forces with different prototypes. It will also help in understanding the kinematics associated with osteosynthesis in a clinical setting. Although this study revealed that the novel-shaped u-HA/PLLA ladder plate system could exert stability immediately after the SSRO osteotomy, all the osteosynthesis systems showed the unstable conditions of biomechanical intensity. This could be the first step in understanding the behavior of these osteosynthesis devices. In future research, it would be necessary to monitor the clinical stability of various osteosynthetic fixation systems including bioresorbable systems because these devices can change biomechanical properties and intensity with the interaction of the surrounding tissues and the process of bioresorbability. 

In addition, since an effective method for reproducing mandibular function is yet to be developed, we should not assume that the biomechanical results observed in this study would elicit an immediate change in the current application of osteosynthesis methods. Here, it should be noted that the complex oromandibular interactions between the mandible and adjacent musculatures are taken into consideration. However, we believe that the findings of the present study could support and stimulate an active discussion about future clinical applications in orthognathic surgery.

## 5. Conclusions

The u-HA/PLLA ladder plate system significantly optimized the resistance and stability of plate fixation in vitro as compared with a single plate fixing standard SSRO. Due to its bioactive, osteoconductive, as well as bioresorbable properties, this u-HA/PLLA composite fixation system has much potential to be widely and safely applied as a next generation material in orthognathic surgery. 

## Figures and Tables

**Table 1:** Mean values and respective standard deviations for the tensile strength test for each group.

Tensile Strength Evaluation			
**Stress at the Time of 1 mm Move (N)**			
**Plate**	**Single u-HA/PLLA Plate**	**Double u-HA/PLLA Plate**	**u-HA/PLLA Ladder Plate**
#1	152.3	300.4	335.5
#2	161.5	302.8	342.2
#3	149.9	294.5	341.4
Ave.	154.6	299.2	339.7
S.D.	6.1	4.3	3.7
**Maximum Stress (N)**			
**Plate**	**Single u-HA/PLLA Plate**	**Double u-HA/PLLA Plate**	**u-HA/PLLA Ladder Plate**
#1	198.3	394.7	459.1
#2	190.8	443.7	446.4
#3	202.6	428.5	464.4
Ave.	197.2	422.3	456.6
S.D.	6.0	25.1	9.3

**Table 2:** Mean values and respective standard deviations for the shear strength test for each group.

Shear Strength Evaluation			
**Stress at the Time of 1 mm Move (N)**			
**Plate**	**Single u-HA/PLLA Plate**	**Double u-HA/PLLA Plate**	**u-HA/PLLA Ladder Plate**
#1	22.4	46.9	110.2
#2	20.6	50.1	115.1
#3	21.1	48.6	111.8
Ave.	21.4	48.5	112.4
S.D.	1.0	1.6	2.5
**Maximum Stress (N)**			
**Plate**	**Single u-HA/PLLA Plate**	**Double u-HA/PLLA Plate**	**u-HA/PLLA Ladder Plate**
#1	63.8	126.8	187.2
#2	63.3	123.3	174.7
#3	63.0	91.5	171.3
Ave.	63.4	113.9	177.7
S.D.	0.4	19.4	8.4

**Table 3:** Mean values and respective standard deviations for the amount of movement by adding load for each group.

**The Amount of Movement of 50N Load (mm)**				
**Plate**	**Single Titanium Plate**	**Single u-HA/PLLA Plate**	**Double u-HA/PLLA Plates**	**u-HA/PLLA Ladder Plate**
#1	2.58	4.02	1.34	0.80
#2	2.05	3.64	1.16	1.32
#3	1.68	3.33	1.38	1.14
Ave.	2.10	3.66	1.29	1.09
S.D.	0.45	0.35	0.12	0.26
**The Amount of Movement of 130N Load (mm)**				
**Plate**	**Single Titanium Plate**	**Single u-HA/PLLA**	**Double u-HA/PLLA**	**u-HA/PLLA Ladder Plate**
#1	7.39	-	-	3.33
#2	6.67	-	6.21	3.57
#3	7.87	-	5.61	3.49
Ave.	7.31	-	-	3.46
S.D.	0.60	-	-	0.12

## Abbreviations

u-HA	unsintered hydroxyapatite;
PLLA	poly-l-lactide-acid;
SSRO	sagittal split ramus osteotomy
